# Seasonal Shifts in Water Utilization Strategies of Typical Desert Plants in a Desert Oasis Revealed by Hydrogen and Oxygen Stable Isotopes and Leaf δ^13^C

**DOI:** 10.3390/plants15020340

**Published:** 2026-01-22

**Authors:** Yang Wang, Wenze Li, Wei Cai, Nan Bai, Jiaqi Wang, Yu Hong

**Affiliations:** 1 College of Life Sciences and Technology, Inner Mongolia Normal University, Hohhot 010022, China; ywang@imnu.edu.cn (Y.W.);; 2 Key Laboratory of Biodiversity Conservation and Sustainable Utilization of the Mongolian Plateau, Inner Mongolia Autonomous Region, Hohhot 010022, China; 3 College of Geographical Sciences, Inner Mongolia Normal University, Hohhot 010022, China; 4 Key Laboratory of Infinite-Dimensional Hamiltonian Systems and Their Algorithm Applications, Ministry of Education, Hohhot 010022, China

**Keywords:** stable isotopes, water-use strategy, deep soil water, water-use efficiency, desert oasis

## Abstract

Understanding seasonal water acquisition strategies of desert plants is critical for predicting vegetation resilience under increasing hydrological stress in arid inland river basins. In hyper-arid oases, strong evaporative demand and declining groundwater levels impose tightly coupled constraints on plant water uptake across soil–plant–atmosphere continua. In this study, we combined hydrogen and oxygen stable isotopes, Bayesian mixing models, soil moisture measurements and groundwater monitoring, and leaf δ^13^C analysis to quantify monthly water-source contributions and long-term water-use efficiency of three dominant species (*Reaumuria soongarica*, *Tamarix ramosissima*, and *Populus euphratica*) in the Ejina Oasis. Clear ecohydrological niche differentiation was evident among the three species. *R. soongarica* exhibited moderate temporal flexibility by integrating shallow and deep soil water with episodic groundwater use, whereas *T. ramosissima* adopted a vertically integrated and hydraulically plastic strategy combining precipitation, multi-depth soil water, and groundwater. In contrast, *P. euphratica* followed a conservative strategy, relying predominantly on deep soil water with only minor and transient inputs from precipitation and groundwater. Across species and seasons, deep vadose-zone soil water (120–200 cm) consistently acted as the most stable and influential reservoir, buffering seasonal drought and sustaining transpiration. *T. ramosissima* maintained the highest intrinsic water-use efficiency, and *P. euphratica* exhibited consistently lower efficiency associated with sustained access to stable deep soil water. These contrasting strategies reveal multiple pathways of hydraulic stability and plasticity that underpin vegetation persistence under progressive groundwater depletion. By linking water-source partitioning with physiological regulation, this study provides a mechanistic basis for understanding plant water-use strategies and informs ecological water management and species-specific restoration in hyper-arid inland oases.

## 1. Introduction

Desert ecosystems are among the world’s most water-limited environments and are highly sensitive to ongoing climatic warming and hydrological change. Characterized by extremely low precipitation, strong evaporative demand, and highly heterogeneous soil–water availability, these systems impose severe constraints on plant establishment, water acquisition, and physiological functioning [1]. Small variations in water availability can therefore trigger disproportionate ecological responses, including shifts in plant water-use strategies, vegetation structure, and ecosystem functioning. Despite sparse vegetation cover, desert shrublands and riparian forests play critical roles in maintaining biodiversity, stabilizing soils, and regulating land–atmosphere water fluxes [2]. In hyper-arid inland basins, riparian vegetation further serves as a keystone component linking surface water, groundwater, and terrestrial ecosystems. Given that water availability is the principal limiting resource, understanding how desert plants obtain, partition, and utilize water across seasons is fundamental for predicting their responses to intensified drought and groundwater decline [3,4].

Hydrogen and oxygen stable isotopes provide powerful tools for quantifying plant water sources in arid environments. Numerous studies have documented that shrubs and trees adjust their uptake seasonally—accessing shallow soil water following episodic rainfall pulses, then shifting to deeper layers or groundwater as surface moisture is depleted [5,6]. Such flexibility is often interpreted as an adaptive strategy that buffers plants against short-term hydrological variability. The “two water worlds” hypothesis further proposes limited mixing between tightly bound soil water used by plants and mobile water contributing to runoff and recharge [7,8]. While this framework has advanced understanding of plant–soil–water interactions, its applicability to hyper-arid inland basins—where vadose-zone evaporation is intense, infiltration depth is limited, and groundwater recession is persistent—remains uncertain. In these environments, the distinction between shallow soil water, deep vadose-zone moisture, and groundwater may be blurred by strong evaporative enrichment and vertical hydraulic connectivity, complicating interpretations of isotopic signatures. Although δ^2^H–δ^18^O are effective for resolving short-term water-source use, they provide limited insight into longer-term physiological responses to water stress. In contrast, plant water-use efficiency reflects integrated regulation of carbon assimilation and transpiration over longer time scales. Leaf δ^13^C is widely used as a proxy for intrinsic water-use efficiency (iWUE), capturing stomatal behavior and photosynthetic discrimination over days to months [9]. Variations in δ^13^C have been linked to drought tolerance, rooting depth, and hydraulic regulation across diverse ecosystems, including arid shrublands and alpine environments. Recent studies highlight that combining water-source partitioning with δ^13^C-based indicators can reveal coordinated carbon–water regulation strategies that are not apparent from isotope hydrology alone [10,11]. However, few studies have simultaneously examined δ^2^H–δ^18^O-based water-source dynamics and δ^13^C-based iWUE across multiple coexisting species in hyper-arid inland oases, where water availability is tightly constrained and human regulation strongly modifies hydrological processes.

The Ejina Oasis in the lower Heihe River Basin represents a critical but vulnerable hydrological–ecological system that has undergone pronounced groundwater decline and vegetation degradation due to upstream water abstraction [12,13]. Within this oasis–desert transition zone, dominant woody species occupy contrasting ecological niches and collectively structure the riparian and desert vegetation mosaic. *Reaumuria soongarica*, *Tamarix ramosissima*, and *Populus euphratica* represent the major woody life forms in this system, spanning desert shrublands and riparian forests and coexisting under a shared but increasingly stressed groundwater regime. Their contrasting growth forms, rooting architectures, and physiological traits create a natural template for exploring how species differ in water acquisition and regulation under extreme aridity. To address these gaps, this study integrates δ^2^H–δ^18^O isotopes, Bayesian mixing models, soil moisture and groundwater monitoring, and leaf δ^13^C measurements to quantify seasonal water-use strategies and long-term iWUE of three coexisting desert species. Specifically, we aim to answer the following questions: (1) How do these species partition rainfall, shallow, mid-depth, deep soil water, and groundwater across monthly time scales? (2) Does deep soil water function as a key hydrological buffer during periods of groundwater recession? (3) How do seasonal shifts in water-source use correspond to long-term physiological adjustments inferred from leaf δ^13^C? By linking short-term water acquisition with long-term carbon–water regulation, this study provides an integrated understanding of plant water-use strategies and ecohydrological resilience in hyper-arid desert oases undergoing rapid hydrological degradation.

## 2. Results

### 2.1. Seasonal Dynamics of Soil Moisture and Groundwater Depth

**Figure 1:**
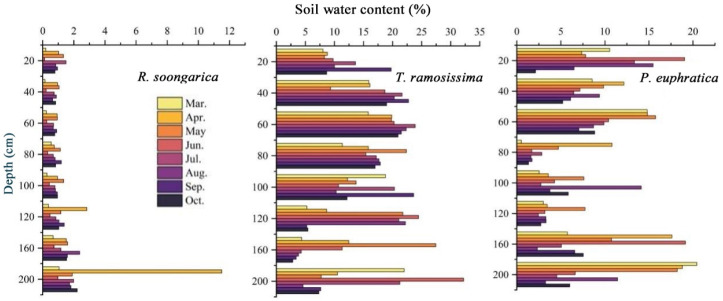
Vertical distribution of gravimetric soil water content (%) along the 0–200 cm soil profile from March to October for *R. soongarica*, *T. ramosissima* and *P. euphratica*. Horizontal bars represent mean soil water content at each depth interval, and colors denote monthly sampling periods.

**Figure 2:**
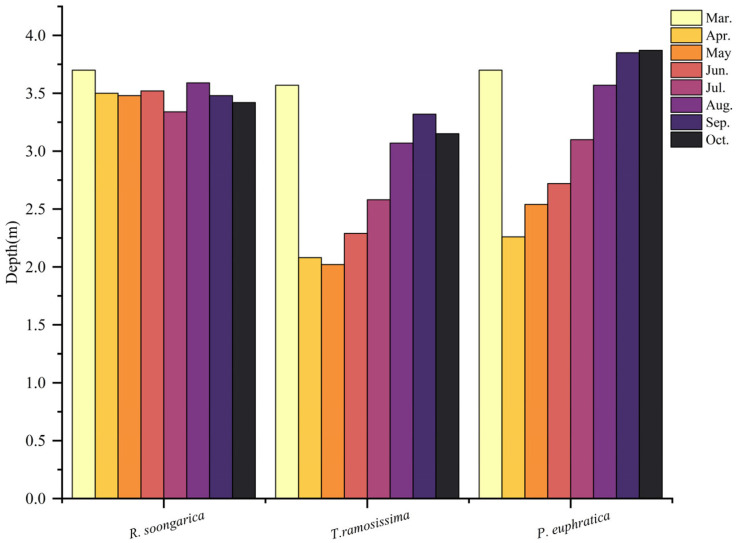
Seasonal variation in groundwater depth from March to October beneath the three dominant desert plant communities. Groundwater depth (cm below ground surface) is shown for *R. soongarica*, *T. ramosissima*, and *P. euphratica*, with greater depths indicating lower groundwater tables.

Soil moisture and groundwater level showed pronounced seasonal and vegetation-specific differences across the desert oasis (Figure 1 and Figure 2). For *R. soongarica*, soil water content remained consistently low (<10%) throughout the 0–200 cm soil profile throughout the study period. Slightly higher moisture occurred in deep soil layers (≥120 cm) during early spring, followed by a gradual decline toward summer, with occasional increases at 160–200 cm in mid to late summer, but without a clear or persistent vertical gradient. Overall, soil moisture in *R. soongarica* plots remained consistently low throughout the profile, with weak vertical gradients and limited seasonal variability. In contrast, *T. ramosissima* showed substantially higher soil water content with clear depth-dependent and seasonal patterns. Moisture increased from shallow soils (0–20 cm) to mid-depth layers (40–120 cm), where values frequently exceeded 20%, particularly during late spring and summer. Deep layers (160–200 cm) showed episodically high moisture with pronounced summer peaks, while shallow soils remained consistently drier and more seasonally variable. Overall, soil moisture was concentrated in mid- to deep-soil layers with distinct seasonal dynamics. This pattern suggests potential buffering effects of deep soil water and groundwater. For *P. euphratica*, soil water content displayed a distinct vertical pattern. Relatively high moisture was persistently observed in deep soil layers (160–200 cm) across the growing season, while shallow layers (0–20 cm) showed only episodic increases during spring and early summer, indicating limited and transient surface recharge. In contrast, intermediate depths (80–120 cm) maintained comparatively low soil water content with limited seasonal variation. Overall, soil moisture for *P. euphratica* was concentrated in deep layers, with weaker and more transient contributions from surface soils, reflecting strong coupling with groundwater dynamics.

*T. ramosissima* maintained the shallowest groundwater levels (generally <3.5 m), followed by *P. euphratica*, while *R. soongarica* occurred in the driest habitats with groundwater consistently >3.5–4 m. Seasonal patterns showed progressive deepening of the groundwater table from spring to late summer, with the strongest drawdown occurring beneath *P. euphratica* and *T. ramosissima*, indicating intensive phreatic extraction during peak transpiration.

### 2.2. Seasonal Variation in δ^18^O Signatures and Water-Source Contributions Among Species

**Figure 3:**
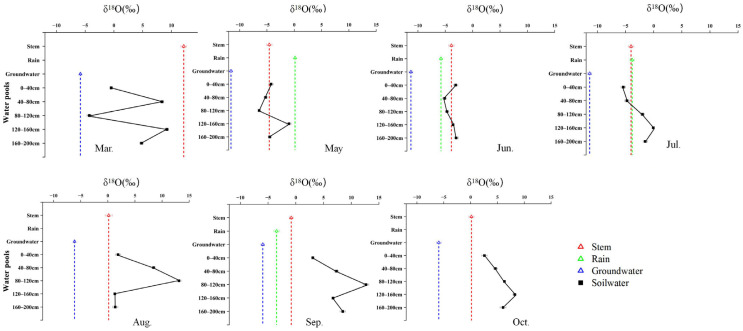
Monthly variation in δ^18^O signatures of different water pools associated with *R. soongarica*, including soil water at different depths, xylem water, groundwater, and precipitation, from March to October.

**Figure 4:**
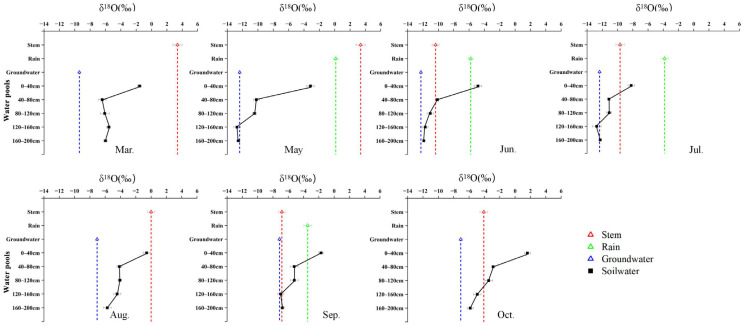
Monthly variation in δ^18^O signatures of different water pools associated with *T. ramosissima*, including soil water at different depths, xylem water, groundwater, and precipitation, from March to October.

**Figure 5:**
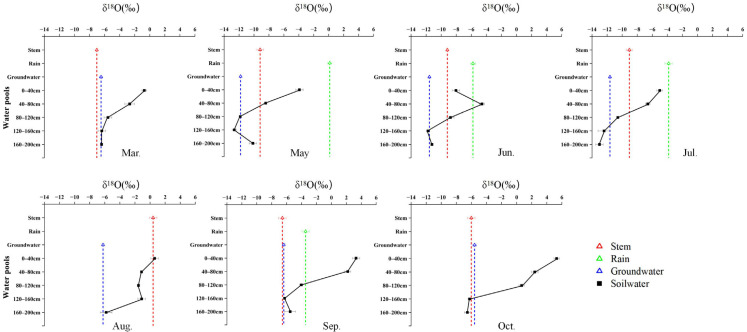
Monthly variation in δ^18^O signatures of different water pools associated with *P. euphratica*, including soil water at different depths, xylem water, groundwater, and precipitation, from March to October.

**Figure 6:**
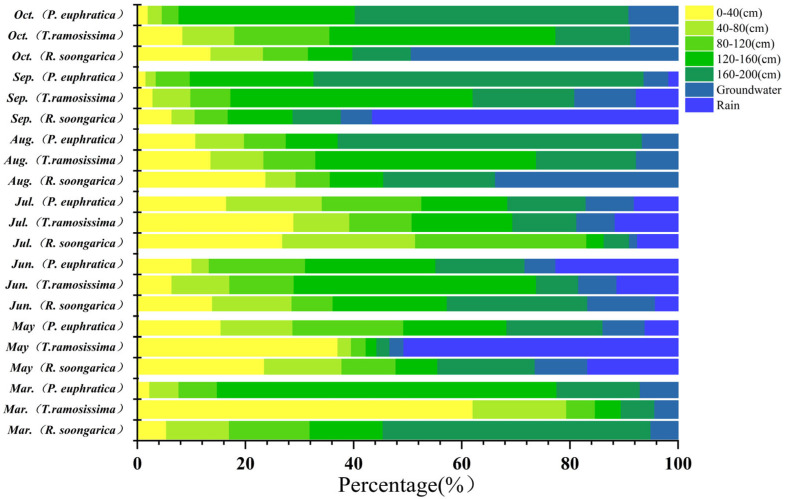
Seasonal variation in the proportional contributions of different water sources (rain, soil water at different depths, and groundwater) to *R. soongarica*, *T. ramosissima*, and *P. euphratica* from March to October, estimated using Bayesian isotope mixing models.

Seasonal water use of the three species was jointly constrained by isotopic patterns and Bayesian mixing model estimates, revealing distinct but seasonally dynamic water-use strategies (Figure 3, Figure 4, Figure 5 and Figure 6). *R. soongarica* relied primarily on deep soil water (160–200 cm; ~50%) in early spring under strong vertical isotopic heterogeneity. By May, reduced isotopic variability coincided with a shift toward mixed use of shallow (0–40 cm) and deep soil water (~42%). During summer (June–August), water use was highly dynamic, alternating among mid–deep soil water in June (~47%), shallow soil water in July (~52%), and multi-layer uptake in August (~66%), accompanied by increased groundwater contribution (~34%). In autumn, uptake shifted toward rainwater in September (~57%) and groundwater in October (~49%), reflecting pronounced seasonal plasticity (Figure 3 and Figure 6). *T. ramosissima* showed pronounced seasonal plasticity. In March, uptake was primarily from shallow soil water (0–40 cm; ~62%). By May, uptake shifted rapidly toward rain-derived sources, with rainwater contributing ~51% and shallow soil water accounting for ~37%, accompanied by minor contributions from deep soil water and groundwater. A multi-source strategy prevailed in summer, with deep soil water (120–160 cm) dominating water uptake in June (~45%), followed by increased reliance on shallow soil water (0–40 cm) in July (~29%), and a renewed dominance of deep soil water (120–200 cm) in August (~60%). In autumn, water uptake increasingly relied on deep soil water and groundwater, with deep soil water contributing ~64% in September and uptake concentrating in the 120–160 cm layer by October (~56%) (Figure 4 and Figure 6).

In contrast, *P. euphratica* predominantly relied on deep soil water throughout the growing season. In March, deep soil layers (120–200 cm) accounted for ~78% of total uptake, coinciding with minimum δ^18^O values at depth. By May, contributions from different soil layers became more evenly distributed (17 ± 3%), while groundwater and precipitation remained minor sources. During summer, precipitation and deep soil water jointly supported transpiration, with rainwater contribution increasing in June (~23%). In August, reliance on the deepest soil layer (160–200 cm) intensified (~56%), despite isotopic intersections occurring only in shallow soil water. In autumn, deep soil water dominated water uptake, contributing ~61% in September and ~84% in October, accompanied by a modest increase in groundwater use (~9%) (Figure 5 and Figure 6).

### 2.3. Seasonal Patterns of Leaf δ^13^C and Inferred Water-Use Efficiency

**Figure 7:**
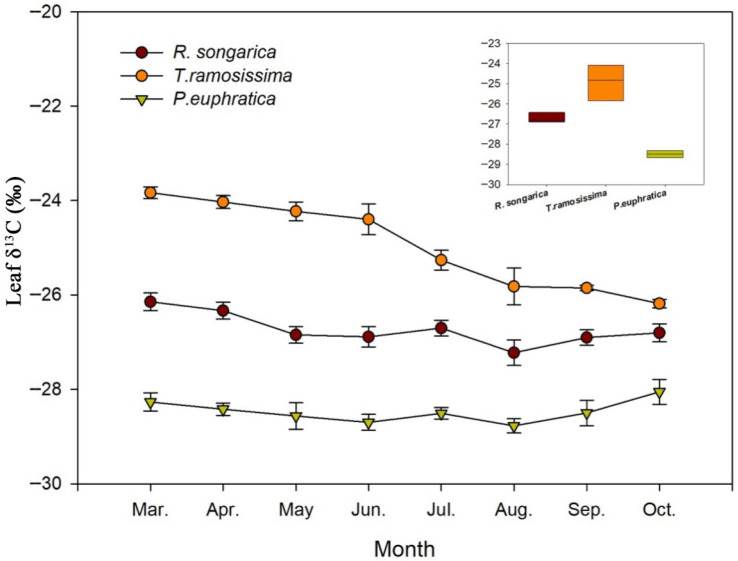
Seasonal variation in leaf δ^13^C values for *R. soongarica*, *T. ramosissima*, and *P. euphratica* from March to October. δ^13^C values reflect integrated physiological responses to water availability and water-source use over the growing season.

Leaf δ^13^C exhibited pronounced species-specific differences and moderate seasonal variation (Figure 7). *T. ramosissima* consistently showed the most enriched δ^13^C values (−25‰ to −26.5‰), indicating the highest iWUE and relatively tight stomatal regulation. *R. soongarica* displayed intermediate δ^13^C values (−26‰ to −27.5‰) with limited seasonal variation, reflecting moderate iWUE and flexible physiological regulation consistent with its variable reliance on mid-depth and deep soil water. In contrast, *P. euphratica* maintained the most depleted δ^13^C (−27.5‰ to −29.5‰) and exhibited minimal seasonal variation, suggesting lower iWUE associated with sustained access to relatively stable deep soil water and reduced stomatal limitation. Across all species, leaf δ^13^C values showed a tendency toward slight depletion during mid-summer, followed by modest enrichment in autumn. This seasonal pattern is consistent with shifts in evaporative demand and plant water status rather than abrupt changes in water source, indicating that leaf δ^13^C integrates longer-term physiological responses to seasonal water availability rather than short-term fluctuations in soil moisture.

## 3. Discussion

### 3.1. Ecohydrological Niche Differentiation and Vertical Water-Source Partitioning

The three dominant species exhibited clear yet contrasting seasonal patterns of water uptake, forming distinct ecohydrological niches through differential use of vertically structured soil water pools. *R. soongarica* displayed a moderate but distinct degree of seasonal flexibility, shifting from dominant deep soil water use in early spring to mixed shallow–deep uptake in late spring, followed by rapid switching among soil layers and episodic groundwater use during summer. Although precipitation exhibits strong seasonality in the study area, with most rainfall occurring during the growing season, the observed shifts in water-source use—particularly for *R. soongarica*—do not reflect a simple rainfall-driven pulse response. Instead, this behavior reflects a highly opportunistic multi-layer strategy in which shallow infiltration, deep vadose-zone water, and groundwater are alternately exploited depending on short-term hydrological conditions [14]. Such dynamic switching allows *R. soongarica* to persist under strong temporal variability in soil moisture typical of hyper-arid deserts, extending previous conceptualizations of shrub water use beyond simple shallow–deep dichotomies [15,16,17]. *T. ramosissima* exhibited pronounced seasonal plasticity but followed a more structured progression. Water uptake shifted rapidly from shallow soil layers in early spring to deeper soil water and groundwater by late spring, and subsequently integrated precipitation and multi-depth soil water during summer. This vertically integrated strategy indicates strong hydraulic connectivity across the soil profile, consistent with *Tamarix* species functioning as flexible exploiters of steep vertical gradients in water availability rather than relying on a single dominant source [18,19]. Although groundwater levels approached ~200 cm during early spring, deep soil moisture increased later in the growing season. This delayed response is consistent with limited capillary rise and time-lagged vertical recharge in coarse-textured desert soils. In contrast, *P. euphratica* showed a markedly conservative strategy, predominantly relying on deep soil water throughout the growing season, with only minor and transient contributions from precipitation and groundwater. Although groundwater input increased slightly in late summer and autumn, deep vadose-zone water remained the dominant source across seasons. This pattern suggests that *P. euphratica* is more tightly coupled to deep soil moisture than directly to the groundwater table, highlighting a rooting and uptake strategy that prioritizes stability over responsiveness [20,21,22]. Together, these contrasting strategies reveal a clear vertical and seasonal partitioning of water resources among coexisting species. Rather than simple separation between groundwater-dependent trees and rainfall-driven shrubs, niche differentiation emerges from differences in uptake stability, vertical integration, and temporal flexibility, which collectively reduce interspecific competition and promote coexistence in desert oasis ecosystems.

### 3.2. Deep Soil Water as a Major Hydrological Reservoir in Hyper-Arid Inland Basins

Across species and seasons, deep soil water within the 120–200 cm profile consistently emerged as a critical source sustaining plant transpiration, underscoring its role as a long-term hydrological reservoir in hyper-arid inland basins. In groundwater-controlled desert oases, seasonal precipitation modulates, rather than governs, plant water-use strategies. The relatively stable and isotopically depleted signatures of deep soil water indicate slow turnover and limited evaporative enrichment, consistent with observations from arid and semi-arid ecosystems worldwide [23,24]. This stability suggests that deep soil water buffers seasonal variability in precipitation, such that even during months with higher rainfall, plant water uptake remains strongly influenced by deep soil water and groundwater, while during dry periods these deeper reservoirs provide continuity of supply. Traditional frameworks often emphasize either direct groundwater uptake by phreatophytes or rapid exploitation of rainfall pulses by shrubs [25]. However, our results demonstrate that deep soil water contributes more persistently and broadly to plant water use than either shallow infiltration or groundwater across much of the growing season. For *P. euphratica*, deep soil water dominated uptake even during early spring when groundwater tables were relatively shallow, suggesting that water uptake in inland poplar forests is closely associated with deep vadose-zone storage rather than direct groundwater use alone [26]. Similarly, *T. ramosissima* increasingly relied on deep soil water during late summer and autumn as upper soil layers became depleted, while *R. soongarica* consistently accessed deep soil water during early spring and again in autumn when shallow moisture was unavailable. These findings highlight the need to explicitly incorporate deep soil water storage into ecohydrological models of inland river basins [27]. Management strategies focused solely on surface runoff or short-term groundwater recovery may therefore underestimate the importance of maintaining recharge and retention of deep soil moisture for sustaining desert vegetation, maintaining deep vadose-zone moisture is essential for stabilizing inland river ecosystems under ongoing hydrological degradation.

Importantly, the capacity of different species to access and utilize deep soil water is fundamentally constrained by belowground root traits, including rooting depth, vertical root distribution, and root plasticity. Previous studies have shown that deep-rooted phreatophytes typically rely on relatively invariant root systems that provide stable access to deep soil water or groundwater, whereas shrubs often develop more plastic, multi-layer root architectures that allow dynamic switching among shallow soil water, deep vadose-zone water, and groundwater in response to hydrological variability [28,29,30]. Such structural differences in root systems provide a mechanistic basis for the contrasting water-source use patterns observed among species in this study and mediate their responses to seasonal water availability.

### 3.3. Water-Use Efficiency, Physiological Adaptation and Implications for Vegetation Resilience

Integrating leaf carbon isotope evidence provides insight into how contrasting water-use strategies translate into long-term physiological adaptation [31]. The consistently depleted leaf δ^13^C values of *P. euphratica* indicate relatively low iWUE, reflecting sustained access to deep and stable soil water pools rather than frequent stomatal regulation in response to short-term moisture variability. While this conservative strategy minimizes short-term water stress, it also implies limited physiological flexibility under progressive deep-soil drying or groundwater decline, increasing vulnerability under continued hydrological degradation [32]. *T. ramosissima* exhibited enriched δ^13^C values, indicating higher iWUE and tighter stomatal regulation. Coupled with its vertically integrated and seasonally flexible water-use strategy, this physiological trait underpins *Tamarix*’s resilience under fluctuating water availability, helping to explain its persistence and expansion in disturbed river corridors globally [33,34]. *R. soongarica* showed intermediate and seasonally variable δ^13^C values, consistent with flexible physiological regulation supported by opportunistic access to multiple soil water pools rather than direct reliance on precipitation alone. This pattern is indicative of an opportunistic yet regulated strategy supported by flexible root systems, allowing shrubs to exploit transient moisture inputs while maintaining access to deeper and more stable water sources during dry periods [35,36]. This dual strategy enhances resilience under alternating wet and dry conditions and reflects a balance between flexibility and efficiency characteristic of desert shrubs. Taken together, these findings indicate that species with flexible rooting systems and high water-use efficiency are better adapted to increasing aridity, while species tightly linked to deep stable water sources face increasing stress under groundwater decline [37]. These findings have direct implications for ecological water resource management in inland river basins. Management strategies focused solely on surface runoff regulation or short-term groundwater recovery may underestimate the critical buffering role of deep vadose-zone soil water in sustaining desert vegetation. Ecological water conveyance schemes that enhance deep soil moisture recharge—through controlled river flooding, channel infiltration, or reduced vadose-zone drainage—are therefore essential for maintaining vegetation stability under ongoing hydrological degradation [38,39]. Taken together, our results indicate that species with flexible rooting systems and relatively high water-use efficiency are better adapted to increasing aridity and hydrological variability, whereas species tightly dependent on deep, stable water sources face increasing stress under sustained groundwater decline. As hydrological pressure intensifies in the Heihe River Basin, vegetation composition may progressively shift toward more drought-tolerant shrub communities unless groundwater tables and deep soil moisture are actively stabilized. These insights highlight the necessity of ecological water management strategies that integrate groundwater regulation, deep soil moisture conservation, and species-specific habitat requirements to sustain riparian and desert ecosystems in arid inland basins.

## 4. Materials and Methods

### 4.1. Study Site

**Figure 8:**
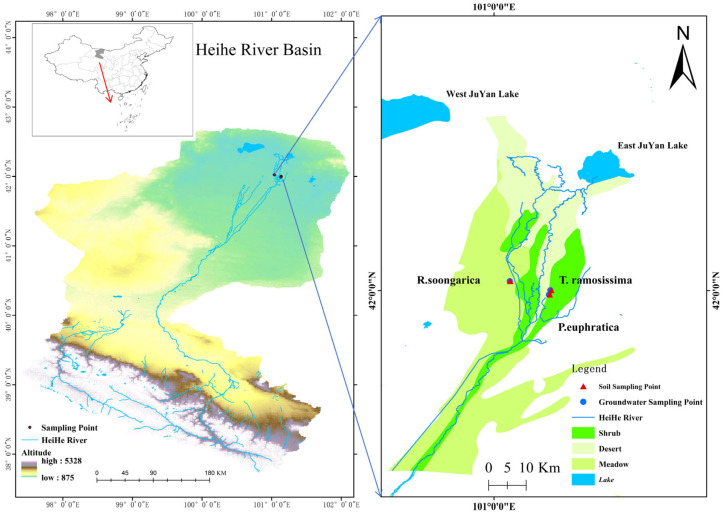
Location of the sampling plots in the Heihe River Basin. Blue arrows indicate the spatial correspondence between the overview map and the enlarged study regions.

The study was conducted in the lower reaches of the Heihe River Basin, within the Ejina Oasis region of northwestern China (approximately 40°20′–42°41′ N, 97°36′–102°08′ E) (Figure 8). This region lies in the interior of the Eurasian continent and is characterized by an extremely arid temperate continental desert climate. Due to its distance from oceanic moisture sources and the blocking effect of surrounding mountain ranges and plateaus, the area receives very limited precipitation. Long-term meteorological records indicate a mean annual precipitation of approximately 34–42 mm, whereas mean annual potential evaporation exceeds 3300 mm. More than 75% of the annual precipitation occurs during the growing season (June–September), with most rainfall events being small (<8 mm) and contributing negligibly to plant water supply. Mean annual air temperature is approximately 8.9 °C, with pronounced seasonal contrasts between cold winters and hot summers. Surface water resources in the region consist mainly of river runoff and lakes, both of which are strongly regulated by upstream water management. In the lower Heihe River Basin, river flow exhibits pronounced seasonal variability driven by ecological water conveyance projects, with increased discharge typically occurring during the vegetation growing season. Groundwater is generally shallow in the oasis zone and deeper in surrounding desert areas, and groundwater dynamics show strong seasonal fluctuations in response to river inflow and channel seepage. River channel seepage constitutes the primary recharge source for groundwater in the study area. Seasonal ecological water conveyance leads to pronounced groundwater table fluctuations, while precipitation infiltration is typically confined to shallow soil layers due to low rainfall and high evaporative demand. As a result, groundwater and groundwater-influenced deep soil water represent the dominant and most reliable water sources for vegetation.

### 4.2. Experiment Design

Three species were selected because they represent the dominant and ecologically contrasting woody life forms in the lower Heihe River Basin. *P. euphratica* is the foundation tree species of desert riparian forests, *T. ramosissima* is a widespread riparian shrub commonly associated with these forests, and *R. soongarica* is a typical desert shrub widely distributed in adjacent arid and hyper-arid landscapes. Three sampling sites were established within a compact area of the Ejina Oasis. The *P. euphratica* woodland site and the *T. ramosissima* scrubland site are located in close proximity on the same side of the river, whereas the site dominated by *R. soongarica* is situated on the opposite bank at a comparable distance from the river channel. Despite differences in bank position, all sites are embedded within the same groundwater-controlled oasis system, and groundwater table depth fluctuated within a comparable range across sites during the study period (Figure 8). Three permanent 50 m × 50 m plots were established at each sampling site. Within each plot, a central soil pit (1 m × 1 m × 2 m) was excavated for stratified soil sampling. Soil layers were defined following standard dryland ecohydrology protocols, with sampling intervals at 0–20, 20–40, 40–60, 60–80, 80–100, 100–120, 120–160, and 160–200 cm. To minimize disturbance effects during repeated monthly sampling, soil samples were collected from freshly exposed soil faces adjacent to previously sampled locations within the pit, avoiding re-sampling of the same surface. After each sampling event, the pit was immediately covered to reduce evaporative losses and heat exchange with the atmosphere. Within a 5 m radius of each pit, three healthy, mature individuals of each target species were selected for xylem water extraction.

### 4.3. Soil Moisture and Plant and Groundwater Sampling

Sampling was conducted around the middle of each month during the growing season from March to October 2021 to capture representative monthly hydrological conditions and to avoid transient isotope signals associated with short-term rainfall or river recharge events. Fresh mass was recorded immediately in sealed aluminum containers to prevent evaporation. Soil water content in this study was expressed as gravimetric soil moisture (%). Samples were oven-dried at 105 °C for 8 h, and gravimetric soil moisture was calculated as:

(1) $$W=\frac{W_{1}-W_{2}}{W_{2}-W_{0}}\times 100\%$$

where $W_{0}\;$ is container mass, $W_{1}$ is wet mass plus container, and $W_{2}$ is dry mass plus container. This approach allows high accuracy under strong evaporative gradients typical of arid regions.

Xylem samples were collected from 1–2 cm diameter terminal branches during early morning to minimize evaporative enrichment. Bark and phloem were removed to expose fresh sapwood, which was immediately sealed in 50 mL borosilicate vials using Parafilm^®^ (Daehan Scientific Co., Ltd., Wonju, Korea) to prevent isotopic alteration. All samples were stored in insulated coolers and transferred to a −20 °C freezer within four hours, following best practices recommended for isotope hydrology [13]. Groundwater was accessed by augering to the water table and installing a PVC pipe as a temporary well. During sampling, a pre-cleaned sampling bottle was tied with a thin nylon line and lowered directly into the groundwater inside the PVC pipe. The bottle was gently moved to allow adequate mixing before retrieval. Samples were sealed and stored at 4 °C prior to isotope analysis.

### 4.4. Water Extraction and Isotope Measurement

Soil and xylem water were extracted using cryogenic vacuum distillation at 105 °C for 4 h. All samples were processed following widely validated protocols to minimize evaporative fractionation during extraction. The extracted water was analyzed for hydrogen and oxygen isotope composition using an isotope ratio infrared spectroscopy (IRIS) system (LWIA, DLT-100, Los Gatos Research Inc., Mountain View, CA, USA). Detailed analytical procedures and correction methods are described in [12]. Stable isotope values (δ^2^H and δ^18^O) of soil water, groundwater, and precipitation were used as potential end members in isotope mixing models. Water-source contributions were estimated using the IsoSource model based on isotopic mass balance constraints. Isotope ratios were reported relative to Vienna Standard Mean Ocean Water (V-SMOW) as:

(2) $$\delta X\;=\left(\frac{R_{\mathrm{sample}}}{R_{V-\mathrm{SMOW}}}-1\right)\times 1000\;‰\;$$

where *X* represents either ^2^H or ^18^O.

Leaf samples were oven-dried at 65 °C, finely ground, and analyzed for carbon isotope composition (δ^13^C) using a Cavity Ring-Down Spectroscopy system (Picarro G2201-i, Picarro Inc., Santa Clara, CA, USA). Carbon isotope values were calculated as:

$$\delta^{13}C_{\mathrm{leaf}}=\left(\frac{R_{\mathrm{sam}}}{R_{\mathrm{std}}}-1\right)\times 1000\;‰$$

where $R_{\mathrm{sam}}$ and $R_{\mathrm{std}}$ denote the ^13^C/^12^C molar ratios of the sample and the Pee Dee Belemnite standard, respectively. Less negative δ^13^C values indicate tighter stomatal regulation and higher intrinsic water-use efficiency (iWUE).

### 4.5. Water-Source Modeling Using MixSIAR

To quantify contributions from multiple water sources, we employed the MixSIAR Bayesian mixing model. Potential sources included rainfall, groundwater, and soil water from five depth intervals: 0–40, 40–80, 80–120, 120–160, and 160–200 cm. This depth resolution aligns with ecohydrological research in drylands, where strong vertical gradients in water availability and isotopic composition commonly occur. No isotopic fractionation was assumed during root uptake. Three Markov Chain Monte Carlo (MCMC) chains were run with 200,000 iterations and 100,000 burn-in, yielding robust posterior distributions for source contributions. Model convergence was verified through Gelman–Rubin statistics and trace diagnostics. Data from April were excluded from subsequent analyses due to sample contamination.

### 4.6. Data Analysis

Data were processed and analyzed using R version 3.1.2 (‘MixSIAR’ package; R Core Team, 2017). All figures, including soil moisture profiles, isotopic vertical profiles, and source contribution diagrams and seasonal variation in leaf δ^13^C were produced using Origin 2022.

## 5. Conclusions

By integrating hydrogen–oxygen isotope tracing with leaf δ^13^C analysis, this study reveals clear ecohydrological niche differentiation among three dominant desert species. Across species and seasons, deep soil water in the vadose zone (120–200 cm) consistently functioned as the most stable and influential water source supporting plant transpiration under declining groundwater conditions. *R. soongarica* exhibited moderate temporal flexibility by dynamically integrating shallow soil water, deep soil water, and episodic groundwater use. *T. ramosissima* exhibited high hydraulic plasticity, combining multiple water sources while maintaining relatively high water-use efficiency. In contrast, *P. euphratica* relied predominantly on deep soil water, with only minor and transient contributions from precipitation and groundwater, reflecting a conservative and stability-oriented strategy. Contrasting water-use strategies among species reflected differences in hydraulic flexibility and physiological regulation, revealing multiple pathways through which stability and plasticity coexist within desert plant communities, with shrubs exhibiting greater seasonal plasticity and trees showing more conservative water-use behavior. The tight linkage between seasonal water-source dynamics and long-term water-use efficiency underscores the critical buffering role of deep vadose-zone moisture in sustaining desert vegetation under intensifying hydrological stress in hyper-arid inland oases.

## Data Availability

The original contributions presented in this study are included in the article. Further inquiries can be directed to the corresponding author.
